# The Role of Social Determinants in Diagnosis Timing for Fetal Care Center-Eligible Conditions: A Scoping Review

**DOI:** 10.3390/diagnostics14141503

**Published:** 2024-07-12

**Authors:** Abigail B. Wilpers, Barbara Eichhorn, Janene Batten, Katie Francis, Amy B. Powne, Shukri Jumale, Kara Hansen, Katherine Kohari, Scott A. Lorch

**Affiliations:** 1 Department of Family and Community Health, School of Nursing, University of Pennsylvania, Philadelphia, PA 19104, USA; awilpers@nursing.upenn.edu; 2 Research Institute, Children’s Hospital of Philadelphia, Philadelphia, PA 19104, USA; 3 Fetal Therapy Nurse Network, Chicago, IL 60611, USA; kathleen.francis@ssmhealth.com (K.F.); abpowne@ucdavis.edu (A.B.P.); shukri.jumale@childrensmn.org (S.J.); klhansen@cmh.edu (K.H.); 4 Fetal Diagnosis and Treatment Centers, UPMC Magee-Womens Hospital, Pittsburgh, PA 15213, USA; 5 Harvey Cushing/John Hay Whitney Medical Library, Yale University, New Haven, CT 06520, USA; janene.batten@yale.edu; 6 St. Louis Fetal Care Institute, SSM Health Cardinal Glennon Children’s Hospital, St. Louis, MO 63104, USA; 7 UC Davis Fetal Care and Treatment Center, UC Davis Health, Sacramento, CA 95817, USA; 8 Midwest Fetal Care Center, Children’s Minnesota, Minneapolis, MN 55404, USA; 9 Fetal Health Center, Children’s Mercy, Kansas City, MO 64108, USA; 10 Department of Obstetrics, Gynecology & Reproductive Sciences, Yale School of Medicine, New Haven, CT 06510, USA; katherine.kohari@yale.edu; 11 Fetal Care Center, Yale New Haven Hospital, New Haven, CT 06510, USA; 12 Division of Neonatology, Children’s Hospital of Philadelphia, Philadelphia, PA 19104, USA; lorch@chop.edu; 13 Perelman School of Medicine, University of Pennsylvania, Philadelphia, PA 19104, USA

**Keywords:** prenatal diagnosis, fetal anomalies, high-risk pregnancy, social determinants of health, fetal therapy, fetal intervention, maternal–fetal surgery, fetal diagnosis

## Abstract

Timely identification of fetal conditions enables comprehensive evaluation, counseling, postnatal planning, and prenatal treatments. This study assessed the existing evidence on how social determinants of health (SDOH) influence diagnosis timing of fetal conditions appropriate for care in fetal care centers (FCCs). Eligible studies were conducted in the U.S. and published in English after 1999. We employed the Healthy People 2020 SDOH framework to categorize and analyze data from 16 studies, where 86% focused solely on congenital heart disease (CHD). Studies primarily focused on individual-level SDOH, with only 36% addressing structural-level factors. A total of 31 distinct indicators of SDOH were identified, with 68% being unique to individual studies. Indicators often varied in definition and specificity. Three studies covered all five SDOH categories in the Healthy People 2020 Framework. Studies revealed varying and often conflicting associations with SDOH indicators, with race and ethnicity being the most explored (100%), followed by socioeconomic status (69%), maternal age (57%), residence (43%), and structural factors (29%). Our findings highlight the need for more comprehensive research, including conditions beyond CHD, and the establishment of consensus on indicators of SDOH. Such efforts are necessary to gain a deeper understanding of the underlying factors driving disparities in fetal diagnosis and treatment.

## 1. Introduction

Approximately 120,000 pregnancies are affected by congenital anomalies annually, accounting for 10% of fetal deaths and the leading cause of infant mortality [1]. Severe anomalies like congenital heart disease (CHD) and spina bifida can cause lifetime disabilities, over 139,000 annual hospitalizations, and $2.6 billion in hospital costs [2]. Timely prenatal detection is crucial, enabling pregnant individuals and their families to understand fetal conditions, make informed care decisions, and access high-risk perinatal care, including specialized fetal care centers (FCCs) [3]. Early detection facilitates referrals to FCCs for advanced multidisciplinary diagnostic assessments, counseling, postnatal care planning, and, in some cases, prenatal treatments such as maternal–fetal surgery [4]. Unfortunately, some severe fetal conditions, especially those diagnosed via ultrasound (US), exhibit high rates of missed prenatal diagnosis [5,6,7]. Evidence suggests that social determinants of health (SDOH) might contribute to this phenomenon, although the extent of this evidence remains to be synthesized [8,9,10]. SDOH focus on the conditions in which people are born, grow, work, live, and age, as well as the broader forces and systems shaping daily life conditions. SDOH can be more important than healthcare or lifestyle choices in influencing an individual’s state of health [11].

Fetal conditions identified primarily through US pose challenges for routine prenatal detection, often demanding the expertise of skilled perinatal clinicians proficient in performing advanced assessments, such as fetal echocardiograms [8]. Access and quality of perinatal care have been linked to SDOH indicators, representing non-medical influences on health outcomes [11]. SDOH health indicators linked to perinatal care and outcomes include individual-level characteristics such as maternal age, race and ethnicity, and indicators of socioeconomic status, as well as structural-level indicators of resource organization, including the amount and distribution of perinatal health services, and health policies such as Medicaid expansion [12].

A number of severe fetal conditions that benefit from prenatal diagnosis, such as congenital diaphragmatic hernia (CDH) and lower urinary tract obstruction (LUTO), exhibit unexplained missed prenatal diagnoses, with reported rates ranging from 20% to 60% for CDH [5,6] and 53% for LUTO [7]. Missed diagnoses are surprising given the Eurofetus Study’s finding that prenatal US is highly effective, with 97% sensitivity and 100% specificity in detecting myelomeningocele (MMC), a severe type of spina bifida amenable to prenatal intervention [13]. However, beyond the controlled study setting, as many as 30% of MMC cases remain undiagnosed until after birth [4,14,15,16,17,18]. Disparities in perinatal MMC treatment suggest potential social determinants of health influencing diagnosis timing, with postnatal care patients disproportionately represented by Medicaid and low-income individuals, contrasting with prenatal repair recipients, who tend to have commercial insurance and a non-Hispanic White background [19,20,21,22]. The origin of these differences, whether from medical factors hindering prenatal treatment or from a failure in prenatal detection due to low-quality care, remains unclear. Insights from studies on missed prenatal CHD diagnoses offer valuable clues, linking delayed detection to SDOH including lower median household income, reliance on public insurance, and limited sonographer availability [8,10,23].

Understanding the common SDOH linked to missed prenatal diagnoses is crucial for ensuring equitable access to timely and effective prenatal care. Through a scoping review, this study aimed to synthesize the existing literature on SDOH’s relationship with prenatal detection of conditions eligible for specialized prenatal care, like those provided by FCCs. Scoping reviews, particularly useful for emerging evidence, offer insights into evidence types and research methodologies within a field, paving the way for more targeted systematic reviews [24].

## 2. Materials and Methods

The search strategy for this scoping review was designed by an expert medical librarian (JB) in consultation with the first author (ABW) and second author (BE), then peer reviewed by a second expert searcher (TM). The databases were searched using both controlled vocabulary and synonymous free text word to capture the two concepts: high-risk pregnancy or prenatal diagnosis, and social determinants of health. The search strategies were adjusted for the syntax appropriate for each database. No limits, such as language or date range, were applied to the search. An expert medical librarian (JB) identified relevant studies by searching OVID Medline(R) ALL (1946 to 16 March 2023), OVID Embase (1974 to 16 March 2023), OVID PsycINFO (1806 to Week 2 March 2023), CINAHL, and Web of Science Core Collection. All searches were conducted on 16 March 2023. Supplementary efforts to identify studies included checking reference lists and contacting experts in the field. The full search strategy is available upon request. Results were uploaded to EndNote (version 20—Clarivate, 1500 Spring Garden Street, Fourth Floor, Philadel-phia, PA 19130, USA) and deduplicated. The final set was uploaded into Covidence systematic review software (Veritas Health Information, Level 10, 446 Collins Street, Melbourne, VIC 3000, Australia) for screening.

We selected studies examining perinatal diagnosis timing for FCC-eligible conditions diagnosed primarily by prenatal US (Table 1). Studies must have assessed whether SDOH were associated with diagnosis timing. SDOH included but were not limited to variables related to maternal age, race, ethnicity, socioeconomic status, education level, relationship status, geographic location, and language spoken. Studies were included if they were published in English, conducted in the United States, and published as a manuscript after 1999. The 1999 Eurofetus study revolutionized prenatal ultrasound (US) by systematically evaluating its accuracy and efficacy in detecting fetal malformations [13]. This research not only provided foundational insights but also sparked a transformative shift in the utilization and standards of prenatal US, making it a landmark for our scoping review. Studies were excluded if they focused solely on genetic syndromes or other conditions that are not primarily detected via US screening, as tests such as cell-free fetal DNA are more universally available and do not rely on advanced screening skills. 

All abstracts and full texts were reviewed by at least 2 authors to ensure rigor (ABW, BE, KF, KH, ABP, SJ). The first and second authors (ABW, BE) reviewed eligibility conflicts and, if needed, discussed them with the research team to reach consensus. Once a set of included texts was finalized, two authors (BE, KF) independently completed data extraction to capture the characteristics and findings of each study, and then compared them to reach consensus. These abstracted data were used to craft Table 2 and the narrative description of the findings across SDOH. We used the Healthy People 2020 SDOH framework, adapted by Elias et al., to organize data into 5 categories of SDOH: (1) Economic Stability, (2) Education, (3) Health and Healthcare, (4) Neighborhood and Built Environment, and (5) Social and Community Context; see the expanded concepts in Table 3 [25]. We listed all indicators used by researchers for each SDOH category and compared across studies. Members of the research team (KF, KH, ABP, SJ, KK) independently reviewed 3 or 4 primary studies each to verify alignment of the original data within the narrative summary.

**Figure 1:**
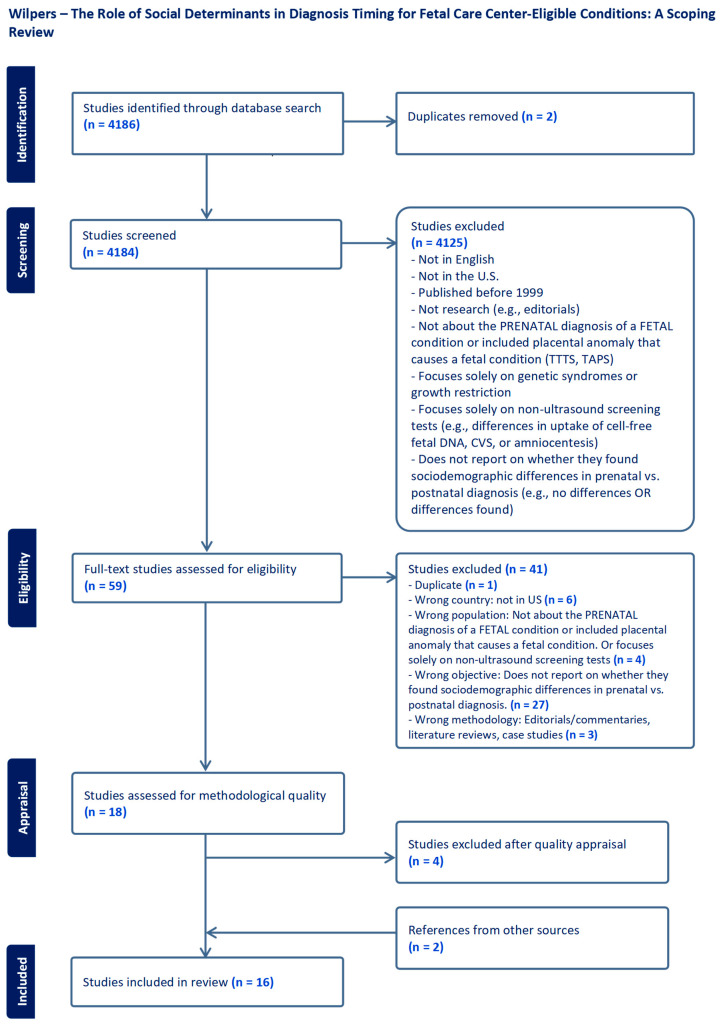
PRISMA flow diagram.

This scoping review was conducted according to the Preferred Reporting Items for Systematic Reviews and Meta-Analyses Extension for Scoping Reviews (PRISMA-ScR) statement (Figure 1) [38].

## 3. Results

We identified 4184 texts for review, 4125 of which were excluded as their title and abstract did not meet inclusion criteria. A total of 59 studies underwent full-text review. To start, we included 14 studies, and by examining their reference lists, we discovered two more eligible studies. A total of 16 manuscripts were included in our final analysis (Figure 1). These studies were published across different years from 2000 to 2023, indicating no discernible increase in frequency over time. Research was conducted in a range of regions across the U.S. Of the sixteen included studies, thirteen were retrospective cohort studies [8,9,10,23,26,27,29,31,32,33,34,35,37], two were prospective cohort studies [28,36], and one was a case control study (Table 2) [30]. Sample sizes ranged from 95 to 7299, with a median of 605. Most of the research in this area focused solely on CHD (86%) [8,9,10,23,26,27,28,29,31,32,33,34,35,36], though one study examined 20 different types of structural fetal conditions [37], and one examined clubfoot [30]. As a result, the remainder of this review will group all studies of fetal-based conditions together and will not divide the results by the type of condition studied. Diagnosis timing was primarily assessed in terms of prenatal versus postnatal diagnosis.

All of the studies in this sample examined individual-level SDOH, while only 36% also explored structural-level factors. We tallied specific indicators within each study and each SDOH category and determined that there was a total of 31 distinct indicators. Of these indicators, 68% were used in only a single study. Similar indicators were also often defined differently or varied in their level of specificity (e.g., individual income vs. income level associated with zip code). Only three studies in this sample could be considered to have addressed all five categories in the adapted Healthy People 2020 SDOH framework [25,30,33,35].

The studies showed highly variable associations between SDOH factors and diagnosis timing, with race and ethnicity being the most examined (100%), followed by socioeconomic status (69%), maternal age (57%), urban or rural residence (43%), and structural factors (29%). 

**Individual-Level SDOH and Indicators**

**Race and ethnicity**. In this sample, all studies investigated the relationship between race and/or ethnicity and diagnosis timing. Significant associations were reported in 44% of the studies [9,10,26,27,29,32,37]. When comparing studies with significant versus non-significant findings, no discernible patterns emerged. However, two methodological concerns were observed that could notably influence these variable findings. 

First, researchers exhibited variation in their approaches to racial and ethnic classification systems. Some studies used broad categories (e.g., White or non-White) [35] combining various racial and ethnic identities, often grouping those outside of Black, White, and Hispanic or non-Hispanic as ‘other’ or “unknown”, which in one study accounted for 58% of the study sample [37]. A limited number of studies included additional related indicators, like preferred language, providing a more comprehensive representation of individual identities. For example, in a retrospective cohort study on 163 CHD patients (2011–2020), Gianelle et al. found a 3.2 times higher likelihood of lacking prenatal diagnosis among Latino patients and a 5.1 times higher likelihood among those with a non-English-preferred language, leading to a 53-week delay in diagnoses for non-English language preferences [9]. 

Second, the racial and ethnic composition across studies exhibited significant variation, with limited reporting on whether and how adequate representation was assessed. Factors such as the racial and ethnic prevalence for each disease group examined or the alignment with regional demographics were rarely given as context. Representation of White individuals ranged from 27% to 80% of samples (median 60), non-Hispanic Black individuals ranged from 4% to 35% (median 13), and Hispanic individuals ranged from 3% to 57% (median 19). Asian individuals were reported as included in only 50% of studies. No other racial or ethnic groups were consistently reported. A lack of adequate diversity in the study sample was listed as a limitation in 50% of studies. It was often unclear whether the categories used to represent the racial and ethnic characteristics of the sample were maintained during data analysis, or if participant grouping was adjusted due to limited representation in certain groups.

**Socioeconomic status**. Similar to the research on race and ethnicity, studies investigating the relationship between SES and diagnosis timing showed a nearly equal split, with 55% reporting significant findings [8,9,10,23,33,34]. Again, the variability in how similar indicators were defined and structured appeared to be a primary contributor to this diversity of the findings, as disease groups, study sample sizes, statistical methodologies, and representation across SES groups were similar across studies with both significant and non-significant findings. 

Studies examining SES using isolated indicators, such as median household income or insurance status, exhibited more variability in their findings. For instance, in a 2013 population-based study of 4348 infants with CHD, Oster et al. found no significant association between neighborhood poverty level and diagnosis timing [32]. In contrast, in a 2020 population-based study of 4702 infants with CHD, Campbell et al. found that higher median household income, which was found to be colinear with neighborhood poverty level, correlated with increased prenatal diagnosis rates [8]. Among studies exploring insurance type [10,23,28,29,33,34,36], sometimes used as an indicator for an isolated SES measure, only one identified a significant association. In this study, private insurance patients were more likely to have prenatal diagnosis of CHD compared to patients with public insurance [34].

Studies employing a standardized composite approach, incorporating multiple SES dimensions and indicators, more consistently found significant associations between SES and diagnosis timing [9,10,33,34]. Three studies [9,10,34] used a previously validated composite measure of SES that combines six variables focused on dimensions of wealth and income, education, and occupation [39]. These studies showed lower SES quartiles associated with decreased prenatal diagnosis rates for certain conditions. One study used the Social Vulnerability Index (SVI), observing higher SVI quartiles associated with late diagnosis [33]. Consistent significant findings across studies using a composite approach may indicate an improved measure of how multiple socioeconomic factors collectively impact diagnosis timing.

**Maternal age**. Eight studies examined whether maternal age was associated with diagnosis timing [26,29,30,31,32,33,35,37]. Among these, five initially identified associations with diagnosis timing in their preliminary analyses [26,30,31,33,35]. However, after adjusted analyses, associations persisted in only three studies [26,30,35].

Each study differed in its approach to the age variable. In studies where significant associations were found, age was treated as a binary variable (≥30 years [26]; ≥35 years [30]) or organized into three groups (<21, 21–34, ≥35 years). For example, Ailes et al. conducted a retrospective study involving 7299 cases from multiple states, showing a positive association between prenatal CHD diagnosis and advanced maternal age (defined as ≥30 years at birth) [26]. Among women ≥30, 19% had CHD detected prenatally, compared to 13% of those <30. Mahan et al. similarly found age ≥30 at conception to be a strong predictor for prenatal clubfoot detection [30].

In contrast, studies where age was not significant after adjustment examined age as either a continuous variable, a category with more than three groups, or did not specify the method of examination. Apart from advanced maternal age thresholds, researchers did not explain their methodological choices regarding maternal age variables.

**Urban or rural residence**. Within this sample, six studies explored associations between urban vs. rural residence and diagnosis timing, primarily focusing on the potential impact of rural residence [8,9,10,23,30,37]. Rural patient representation of the samples typically comprised approximately 20% rural individuals, in line with the U.S. average based on 2020 U.S. Census data [40]. Once more, an equal split emerged, with three studies showing negative associations between rural residence and prenatal diagnosis [10,23,37] and three studies finding no association. 

Despite varying outcomes, both sets of studies shared similarities in disease focus, sample sizes, and temporal scope (all taking place after 2010, except Waller et al., 2000 [37]), suggesting that differences in how studies defined rural residence likely contributed to the observed variation in outcomes. Krishnan et al. utilized the U.S. Department of Agriculture’s Rural–Urban Continuum Codes, while Mahan et al. relied on criteria from the U.S. Census Bureau [10,30]. These two governmental groups employ distinct methodologies and criteria, resulting in imperfect alignment in the classification of rural residence. Even the term ‘rural’ has great variability, as a 2022 scoping review by Childs et al. found 33 federal definitions of the word [41]. Hill et al. focused solely on population density (<500 people/sqmi) based on maternal zip code, whereas Waller et al. encompassed multiple cities within the Lower Rio Grande Valley without delineating specific criteria [23,37]. Additionally, one study merged rural patients with those in medically underserved areas (MUA), utilizing data from the Health Resources and Services Administration [9].

**Structural-Level SDOH and Indicators**. Only four studies examined structural SDOH, with a focus solely on resource allocation. Even within this narrow focus, contradictory findings emerged. Again, assessing the reliability of contrasting results between significant and non-significant study groups proved challenging, as no clear or consistent differences were noted in study limitations in each group. However, akin to individual-level SDOH, significant differences were observed in the measurement and treatment of similar indicators. Peiris et al. studied how far patients had to travel to reach fetal echocardiogram facilities, while Krishan et al. measured distance both in miles and time to cardiac surgical centers [10,34]. Although some locations offering fetal echocardiograms also have cardiac surgical centers, not all do, and vice versa. Distance to facilities providing fetal echocardiograms did not show a significant difference in diagnosis timing, although most patients lived within 30 miles of such centers. Bivariate analysis showed that increased distance to a cardiac surgical center was linked to delayed or no prenatal diagnosis. However, multivariate analysis did not find a significant association. 

In contrast, Campbell et al. utilized location quotients for diagnostic medical sonographers and obstetricians to assess resource distribution’s impact on diagnosis timing [8]. A lower number of sonographers was linked to decreased rates of prenatal diagnosis, although the number of obstetricians did not affect diagnosis timing. Finally, as noted in the section above, Gianelle et al. combined rural patients with those in MUAs, which represent insufficient primary care health services, and found no association with diagnosis timing [9]. In summary, researchers not only investigated the distribution of different types of resources, including distinct advanced services (as explored by Peiris and Krishnan) and primary care services (as investigated by Campbell and Gianelle), but also demonstrated varying approaches in the indicators and methodologies employed, even when examining the same types of resources.

## 4. Discussion

By examining the research landscape regarding SDOH and their impact on the timing of ultrasound-based detection for fetal conditions, we discovered a scarcity of studies addressing this issue comprehensively. Despite citing frameworks such as Healthy People 2020, which emphasize the multifaceted nature of health determinants, a significant portion (71%) of the studies in this sample focused exclusively on individual factors, neglecting the broader organizational, community, and policy-level influences emphasized in these frameworks [42]. These individual SDOH indicators were also distinct, where 68% were used in only a single study. Only three studies in this sample could be considered to have addressed all five categories outlined in the adapted Healthy People 2020 SDOH framework [25,30,33,35]. Our findings highlight the necessity for more comprehensive research and the establishment of consensus on key indicators of SDOH. This is crucial for gaining a deeper understanding of the underlying factors driving disparities in fetal diagnosis and treatment. 

The quantity of studies included in this review reaffirms the significance of this topic. However, it is important to note that only one SDOH indicator, namely an individual’s race and ethnicity identity, was consistently assessed across all studies. This indicator represents a multitude of complex SDOH constructs, and there was considerable variability in how it was categorized and treated in methodologies. This variability indicates a lack of consensus on the indicators to prioritize and how to format them to measure SDOH categories. This mirrors broader challenges not only within perinatal care, but also across various healthcare sectors and other industries. In a review conducted in 2019, researchers systematically examined the growing array of resources available for measuring SDOH across various sectors [25]. Significant variability was observed in the SDOH categories covered by each tool, with minimal consensus regarding the specific indicators utilized to measure these categories. Of these indicators, 75% were used in only a single SDOH measurement tool. Fewer than one in four of the measurement tools incorporated all SDOH categories, with social/community context, transportation/infrastructure, food environment, and safety as the least likely to be included. Unfortunately, tools tailored for the healthcare sector covered the fewest SDOH categories and the “Health and Healthcare” category had the most unique indicators used only once. Although achieving complete consensus on indicators across tools and studies is not likely or practical, a near-complete absence of agreement impedes the comparison of findings. In the nascent stage of health equity research in fetal diagnosis and treatment, where reporting sociodemographic characteristics of study participants is still evolving [43], starting this conversation now can establish common standards and methodologies. This fosters collaboration and advances health equity initiatives. 

Our findings highlight a significant imbalance in health equity research focus, particularly between CHD diagnosis and other fetal conditions benefiting from prenatal detection. While other conditions may be less prevalent and, in some instances, easier to diagnose via ultrasound than CHD, many of these conditions are not uncommon, are equally severe, and can be prone to missed prenatal diagnoses. For example, neural tube defects like MMC are the second most common fetal anomalies after CHD, and up to 30% of cases go undiagnosed until after birth for unclear reasons, depriving these patients of the option for prenatal intervention [16,17].

This study must be understood in the context of the following limitations. We may not have identified all eligible studies if their SDOH or fetal anomaly identifiers fell outside our search strategy, or if they explored SDOH as part of their analysis but did not report these specific findings in their abstracts. Due to an insufficient number of studies providing baseline sample characteristics, we opted against including assessments of sample representation, such as participation-to-prevalence ratios, in our study design [43]. In addition, only 44% of the sample reported that their datasets included cases that may have resulted in pregnancy termination, fetal demise, or stillbirth, potentially impacting the comprehensive representation of affected populations for these conditions in the literature [10,26,27,28,33,35,37].

## 5. Conclusions

The existing literature fails to reliably explain the role of SDOH in missed prenatal detection of fetal conditions eligible for specialized prenatal care at FCCs. The contrasting findings in similar studies likely stem from variability in SDOH indicators. The research focus imbalance between CHD and other fetal conditions underscores the need for equitable attention across all conditions. By initiating discussions around common and rigorous methodologies, this study lays the foundation for advancements in health equity within fetal diagnosis and treatment. This includes fostering collaboration among researchers, individuals with lived experience, clinicians, advocacy organizations, and policymakers to develop unified approaches.

## Figures and Tables

**Table 1:** Eligible conditions for scoping review.

Congenital Heart Disease (CHD)	Congenital Fetal Anomalies	Complications of Monochorionic Twins	Fetal Anemia
Hypoplastic left heart syndrome (HLHS) Total anomalous pulmonary venous return (TAPVR) Tetralogy of Fallot (TOF) Transposition of the great arteries (TGA) Double inlet left ventricle (DILV) Atrioventricular septal defect (AVSD) Coarctation of the aorta (CoA) Double outlet right ventricle (DORV)	Congenital diaphragmatic hernia (CDH) Myelomeningocele (MMC) Ventral wall defects (e.g., gastroschisis) Lower urinary tract obstruction (LUTO) Sacrococcygeal teratoma (SCT) Congenital cystic adenomatoid malformation (CCAM) Congenital pulmonary airway malformation (CPAM)	Twin-to-twin transfusion syndrome (TTTS) Twin anemia polycythemia sequence (TAPS) Twin reversed arterial perfusion (TRAP) Selective fetal growth restriction (sFGR)	Alloimmunization Non-immune hydrops Parvovirus B19 infection

**Table 2:** Characteristics of the study sample.

Study *Location*	Design Data Sources *as Reported*	Sample Size *	Anomaly Category	Overall PD Rate	Conditions with Highest PD (%)	Conditions with Lowest PD (%)	SDOH and Indicators’ Associations with Diagnosis Timing
Ailes et al., 2014 [26] *Multiple states*	Retrospective cohort National Birth Defects Prevention Study (NBDPS), medical record review, patient self-reported	7299	CHD	15%	HLHS (53)	TAPVR (0.8)	Associated Maternal age Race and ethnicity Not associated Education level
Campbell et al., 2020 [8] *Multiple states*	Retrospective population-based study Medicaid analytic extract (MAX) dataset, claims with maternal–infant linkage, diagnosis code search	4702	CCHD	28%	Not specified	Not specified	Associated Sonographer labor quotient ZIP code level median household income Not associated Ethnicity OB location quotient Race Rural urban score
Evans et al., 2011 [27] *Nevada*	Retrospective cohort Clark County fetal and congenital cardiac databases, surname ethnicity (e.g., Spanish), self-reported ethnicity	327	TOF TGA	<2007: <5% ‘07-‘09: 27%	TGA (33)	TOF (28)	Associated Ethnicity (2007–2009) Not associated Not specified
Friedberg et al., 2009 [28] *California*	Prospective cohort Northern California referral centers, medical record review, parent-completed questionnaires	309	CHD	36%	Heterotaxy (82) Single ventricle (64) HLHS (61)	TAPVR (0) d- & l-TGA (19) Left-heart obstructive lesions (excluding HLHS) (23)	Not associated Ethnicity Household income Maternal employment level Maternal insurance structure Parental education level US provider type
Gianelle et al., 2023 [9] *Maryland*	Retrospective cohort Society for Thoracic Surgery Congenital Heart Disease Database (U of MD center data), medical record review, US Census	163	CHD	75%	Not specified	Not specified	Associated Ethnicity Preferred language Maternal insurance Neighborhood SES quartiles ^‡^ Not associated Race Residence in rural or MUA
Hill et al., 2015 [23] *Wisconsin*	Retrospective cohort Children’s Hospital of Wisconsin, medical record review, US census	535	CCHD	61%	Tricuspid atresia (100) DILV (95) AVSD (85)	TAPVR (7) Pulmonary stenosis (38) CoA (38)	Associated Percent below poverty Rural vs. non-rural Not associated Ethnicity Insurance type Marital status Race
Krishnan et al., 2021 [10] ^¶^ *Multiple states*	Retrospective cohort Fetal Heart Society Research Collaborative, US census, medical record review	1862	HLHS, TGA	79%	HLHS (92)	TGA (58)	Associated Ethnicity (*TGA only*) Lower socioeconomic quartile Rural residence (*TGA only*) Not associated Distance and driving time from a cardiac surgical center Insurance type Race
Liberman et al., 2023 [29] *Massachusetts*	Retrospective cohort Massachusetts Birth Defect Monitoring Program, diagnosis code search, medical record review	1524	CCHD	63%	HLHS (92) Single ventricle (96) Tricuspid atresia (91)	TAPVR (24) CoA (46) d-TGA (70)	Associated with “timely diagnosis” (*prenatal or before hospital discharge*) Rural vs. non-rural residence Not associated Education Ethnicity Insurance type Maternal age Race
Mahan et al., 2014 [30] *Massachusetts, New York, North Carolina*	Case control Slone Epidemiology Center (U of Boston) study data, medical record review, parent interviews	676	Clubfoot	62%	Bilateral clubfoot (71)	Unilateral (54)	Associated Maternal age Race and ethnicity Geography (state) Not associated Education level Number in household Income level Rural vs. urban residence Marital status Employment status
Mozumdar et al., 2020 [31] *New York*	Retrospective cohort Fetal database (center data), medical record review	222	Major CHD ^§^	92%	Not specified	Not specified	Associated Maternal age Not associated Ethnicity Race Interpreting physician experience
Oster et al., 2013 [32] *Georgia*	Retrospective cohort Metropolitan Atlanta Congenital Defects Program (MACDP), diagnosis code search, record review	4348	CHD	10%	HLHS (50)	TAPVR (2%)	Associated Race and ethnicity Hispanic Non-Hispanic Black Non-Hispanic White “Others” Not associated Maternal age Neighborhood poverty level
Perez et al., 2022 [33] *Massachusetts*	Retrospective cohort Boston Children’s Hospital, Partners Healthcare System, diagnosis code search, medical record review	441	CHD	Sample included PDs only and examined early (<24 weeks GA) vs. late diagnosis (21%)	NA	NA	Associated PD ≥ 24 weeks GA Social vulnerability quartile Religion Not associated Ethnicity Insurance type Marital status Maternal age Race
Peiris et al., 2009 [34] *Massachusetts*	Retrospective cohort Boston Children’s Hospital, hospital medical record review, electronic patient care databases	444	CCHD	50%	HLHS (75)	TGA/IVS (27)	Associated Insurance type Socioeconomic position Not associated Distance to fetal echocardiography Race
Pinto et al., 2012 [35] *Utah*	Retrospective cohort Utah Birth Defects Network (CHD cases), US census	1474	Major CHD	39%	Single ventricle (100) DORV (89) Hypoplastic right ventricle (79)	Aortopulmonary windows (0) TAPVR (6) TGA (14)	Not associated Census-tract level education level Census-tract level poverty level Census-tract level rural/urban residence Initiation of prenatal care Education level Maternal age Race
Sekar et al., 2013 [36] *Cincinnati*	Prospective cohort Cincinnati and 8-county surrounding area, record review, parent questionnaire	95	Major CHD	43%	Single ventricle (77) Heterotaxy (66) Complete atrioventricular canal (56)	TAPVR (0) Semilunar valve abnormalities (0) VSD (18)	Not associated Education level Ethnicity Family income bracket Insurance type Race
Waller et al., 2000 [37] *Texas*	Retrospective cohort Texas Birth Defects Monitoring Program data	852	23 categories of birth defects	33%	Anencephaly (71) Encephalocele (63) Gastroschisis (64)	TOF (0) Microcephaly (3) Cleft palate (5)	Associated Race and ethnicity Geographic location Not associated Maternal age

If studies used univariate and multivariate analyses, only the multivariate are presented here. * The majority of studies excluded CHD co-occurring with genetic conditions as these would influence likelihood of detection. However, a small handful of studies (Krishnan, Oster, Friedberg, etc.) did not exclude these conditions. ^§^ Defined as the expected need for intervention within the first year of life. ^‡^ A composite SES score was calculated with factor analysis based on 6 SES variables associated with each block group as previously described by Diez Roux. ^¶^ In these studies, a small fraction (<15%) of the sample included data from Canada, which were excluded from sociodemographic analysis but included in overall PD rates. HLHS—hypoplastic left heart syndrome; TAPVR—total anomalous pulmonary venous return; CCHD—critical congenital heart defect (In Peiris et al., 2009 [34] and Hill et al., 2015 [23] defined as infants who require surgical or transcatheter intervention during the first month of life. Liberman et al., 2023 [29] defined as conditions that require treatment and may cause death in the first year of life. Not explicitly defined in Campbell et al., 2020 [8]); TOF—tetralogy of Fallot; TGA—transposition of the great arteries; d-TGA—dextro (right)-transposition of the great arteries; l-TGA—levo (left)-transposition of the great arteries; MUA—medically underserved area; DILV—double inlet left ventricle; AVSD—atrioventricular septal defect; CoA—coarctation of the aorta; TGA/IVS—transposition of the great arteries with intact ventricular septum; DORV—double outlet right ventricle.

**Table 3:** Healthy People 2020 framework.

Healthy People 2020 SDOH Framework—5 Categories of SDOH	
(1) Economic Stability	Employment Food insecurity Housing stability Poverty
(2) Education	Early childhood education Enrollment in higher education High school graduation Language and literacy
(3) Health and Healthcare	Access to healthcare Access to primary care Health literacy
(4) Neighborhood and Built Environment	Access to healthy foods Crime and violence Environmental conditions Housing quality
(5) Social and Community Context	Discrimination Incarceration Social cohesion

## Data Availability

Data sharing is not applicable to this article as no new data were created or analyzed in this study.

## References

[1] Centers for Disease Control and Prevention Birth Defects. https://www.cdc.gov/birth-defects/about/index.html.

[2] Russo C.A., Elixhauser A. (2007). Hospitalizations for Birth Defects, 2004. Healthcare Cost and Utilization Project (HCUP) Statistical Briefs.

[3] Quartermain M.D., Hill K.D., Goldberg D.J., Jacobs J.P., Jacobs M.L., Pasquali S.K., Verghese G.R., Wallace A.S., Ungerleider R.M. (2019). Prenatal Diagnosis Influences Preoperative Status in Neonates with Congenital Heart Disease: An Analysis of the Society of Thoracic Surgeons Congenital Heart Surgery Database. Pediatr. Cardiol..

[4] Baschat A.A., Blackwell S.B., Chatterjee D., Cummings J.J., Emery S.P., Hirose S., Hollier L.M., Johnson A., Kilpatrick S.J., Luks F.I. (2022). Care Levels for Fetal Therapy Centers. Obstet. Gynecol..

[5] Sperling J.D., Sparks T.N., Berger V.K., Farrell J.A., Gosnell K., Keller R.L., Norton M.E., Gonzalez J.M. (2018). Prenatal Diagnosis of Congenital Diaphragmatic Hernia: Does Laterality Predict Perinatal Outcomes?. Am. J. Perinatol..

[6] Akinkuotu A.C., Cruz S.M., Cass D.L., Cassady C.I., Mehollin-Ray A.R., Williams J.L., Lee T.C., Ruano R., Welty S.E., Olutoye O.O. (2015). Revisiting outcomes of right congenital diaphragmatic hernia. J. Surg. Res..

[7] Malin G., Tonks A.M., Morris R.K., Gardosi J., Kilby M.D. (2012). Congenital lower urinary tract obstruction: A population-based epidemiological study. BJOG.

[8] Campbell M.J., Lorch S., Rychik J., Quartermain M.D., Passarella M., Groeneveld P.W. (2021). Socioeconomic barriers to prenatal diagnosis of critical congenital heart disease. Prenat. Diagn..

[9] Gianelle M., Turan S., Mech J., Chaves A.H. (2023). The Impact of Neighborhood Socioeconomic Status, Race and Ethnicity, and Language on Prenatal Diagnosis of CHD. Pediatr. Cardiol..

[10] Krishnan A., Jacobs M.B., Morris S.A., Peyvandi S., Bhat A.H., Chelliah A., Chiu J.S., Cuneo B.F., Freire G., Hornberger L.K. (2021). Impact of Socioeconomic Status, Race and Ethnicity, and Geography on Prenatal Detection of Hypoplastic Left Heart Syndrome and Transposition of the Great Arteries. Circulation.

[11] World Health Organization Social Determinants of Health. https://www.who.int/health-topics/social-determinants-of-health#tab=tab_1.

[12] Crear-Perry J., Correa-de-Araujo R., Lewis Johnson T., McLemore M.R., Neilson E., Wallace M. (2021). Social and Structural Determinants of Health Inequities in Maternal Health. J. Womens Health.

[13] Grandjean H., Larroque D., Levi S. (1999). The performance of routine ultrasonographic screening of pregnancies in the Eurofetus Study. Am. J. Obstet. Gynecol..

[14] Sacco A., Simpson L., Deprest J., David A.L. (2018). A study to assess global availability of fetal surgery for myelomeningocele. Prenat. Diagn..

[15] Moon-Grady A.J., Baschat A., Cass D., Choolani M., Copel J.A., Crombleholme T.M., Deprest J., Emery S.P., Evans M.I., Luks F.I. (2017). Fetal Treatment 2017: The Evolution of Fetal Therapy Centers—A Joint Opinion from the International Fetal Medicine and Surgical Society (IFMSS) and the North American Fetal Therapy Network (NAFTNet). Fetal Diagn. Ther..

[16] Racusin D.A., Villarreal S., Antony K.M., Harris R.A., Mastrobattista J., Lee W., Shamshirsaz A.A., Belfort M., Aagaard K.M. (2015). Role of Maternal Serum Alpha-Fetoprotein and Ultrasonography in Contemporary Detection of Spina Bifida. Am. J. Perinatol..

[17] Boyd P.A., DeVigan C., Khoshnood B., Loane M., Garne E., Dolk H., EUROCAT Working Group (2008). Survey of prenatal screening policies in Europe for structural malformations and chromosome anomalies, and their impact on detection and termination rates for neural tube defects and Down’s syndrome. BJOG.

[18] Boyd P.A., Wellesley D.G., De Walle H.E., Tenconi R., Garcia-Minaur S., Zandwijken G.R., Stoll C., Clementi M. (2000). Evaluation of the prenatal diagnosis of neural tube defects by fetal ultrasonographic examination in different centres across Europe. J. Med. Screen..

[19] Shao B., Chen J.S., Kozel O.A., Tang O.Y., Amaral-Nieves N., Sastry R.A., Watson-Smith D., Monteagudo J., Luks F.I., Carr S.R. (2023). Postnatal Myelomeningocele Repair in the United States: Rates and Disparities Before and After the Management of Myelomeningocele Study Trial. Neurosurgery.

[20] Foy A.B., Sawin K.J., Derflinger T., Heffelfinger A.K., Koop J.I., Cohen S.S., Sherburne E.C. (2021). Sociodemographic disparities in fetal surgery for myelomeningocele: A single-center retrospective review. J. Neurosurg. Pediatr..

[21] Harbert A.L., Barnett R.R., Abumoussa A.L., Goodnight W.H., Tolleson-Rinehart S., Quinsey C.S. (2022). Sociodemographic disparities as a determinant of fetal versus postnatal surgical myelomeningocele repair. J. Neurosurg. Pediatr..

[22] Best B.J., Cabacungan E.T., Cohen S.S., Kim I., Sherburne E.C., Sawin K.J., Roach A., Foy A.B. (2023). Trends in the early care of infants with myelomeningocele in the United States 2012–2018. Childs Nerv. Syst..

[23] Hill G.D., Block J.R., Tanem J.B., Frommelt M.A. (2015). Disparities in the prenatal detection of critical congenital heart disease. Prenat. Diagn..

[24] Munn Z., Peters M.D.J., Stern C., Tufanaru C., McArthur A., Aromataris E. (2018). Systematic review or scoping review? Guidance for authors when choosing between a systematic or scoping review approach. BMC Med. Res. Methodol..

[25] Elias R.R., Jutte D.P., Moore A. (2019). Exploring consensus across sectors for measuring the social determinants of health. SSM Popul. Health.

[26] Ailes E.C., Gilboa S.M., Riehle-Colarusso T., Johnson C.Y., Hobbs C.A., Correa A., Honein M.A., National Birth Defects Prevention Study (2014). Prenatal diagnosis of nonsyndromic congenital heart defects. Prenat. Diagn..

[27] Evans W.N., Acherman R.J., Castillo W.J., Restrepo H. (2011). The changing occurrences of tetralogy of Fallot and simple transposition of the great arteries in Southern Nevada. Cardiol. Young.

[28] Friedberg M.K., Silverman N.H., Moon-Grady A.J., Tong E., Nourse J., Sorenson B., Lee J., Hornberger L.K. (2009). Prenatal detection of congenital heart disease. J. Pediatr..

[29] Liberman R.F., Heinke D., Lin A.E., Nestoridi E., Jalali M., Markenson G.R., Sekhavat S., Yazdy M.M. (2023). Trends in Delayed Diagnosis of Critical Congenital Heart Defects in an Era of Enhanced Screening, 2004–2018. J. Pediatr..

[30] Mahan S.T., Yazdy M.M., Kasser J.R., Werler M.M. (2014). Prenatal screening for clubfoot: What factors predict prenatal detection?. Prenat. Diagn..

[31] Mozumdar N., Rowland J., Pan S., Rajagopal H., Geiger M.K., Srivastava S., Stern K.W. (2020). Diagnostic Accuracy of Fetal Echocardiography in Congenital Heart Disease. J. Am. Soc. Echocardiogr..

[32] Oster M.E., Kim C.H., Kusano A.S., Cragan J.D., Dressler P., Hales A.R., Mahle W.T., Correa A. (2014). A population-based study of the association of prenatal diagnosis with survival rate for infants with congenital heart defects. Am. J. Cardiol..

[33] Perez M.T., Bucholz E., Asimacopoulos E., Ferraro A.M., Salem S.M., Schauer J., Holleman C., Sekhavat S., Tworetzky W., Powell A.J. (2022). Impact of maternal social vulnerability and timing of prenatal care on outcome of prenatally detected congenital heart disease. Ultrasound Obstet. Gynecol..

[34] Peiris V., Singh T.P., Tworetzky W., Chong E.C., Gauvreau K., Brown D.W. (2009). Association of socioeconomic position and medical insurance with fetal diagnosis of critical congenital heart disease. Circ. Cardiovasc. Qual. Outcomes.

[35] Pinto N.M., Keenan H.T., Minich L.L., Puchalski M.D., Heywood M., Botto L.D. (2012). Barriers to prenatal detection of congenital heart disease: A population-based study. Ultrasound Obstet. Gynecol..

[36] Sekar P., Heydarian H.C., Cnota J.F., Hornberger L.K., Michelfelder E.C. (2015). Diagnosis of congenital heart disease in an era of universal prenatal ultrasound screening in southwest Ohio. Cardiol. Young.

[37] Waller D.K., Pujazon M.A., Canfield M.A., Scheuerle A.E., Byrne J.L. (2000). Frequency of prenatal diagnosis of birth defects in Houston, Galveston and the Lower Rio Grande Valley, Texas 1995. Fetal Diagn. Ther..

[38] Tricco A.C., Lillie E., Zarin W., O’Brien K.K., Colquhoun H., Levac D., Moher D., Peters M.D., Horsley T., Weeks L. (2018). PRISMA Extension for Scoping Reviews (PRISMA-ScR): Checklist and Explanation. Ann. Intern. Med..

[39] Roux A.V., Merkin S.S., Arnett D., Chambless L., Massing M., Nieto F.J., Sorlie P., Szklo M., Tyroler H.A., Watson R.L. (2001). Neighborhood of residence and incidence of coronary heart disease. N. Engl. J. Med..

[40] United States Census Bureau Nation’s Urban and Rural Populations Shift Following 2020 Census. Updated 4 April 2024. https://www.census.gov/newsroom/press-releases/2022/urban-rural-populations.html.

[41] Childs E.M., Boyas J.F., Blackburn J.R. (2022). Off the beaten path: A scoping review of how ‘rural’ is defined by the U.S. government for rural health promotion. Health Promot. Perspect..

[42] Hoyer D., Dee E. (2022). Using Healthy People as a Tool to Identify Health Disparities and Advance Health Equity. J. Public Health Manag. Pract..

[43] Wilpers A., Lynn A.Y., Eichhorn B., Powne A.B., Lagueux M., Batten J., Bahtiyar M.O., Gross C.P. (2022). Understanding Sociodemographic Disparities in Maternal-Fetal Surgery Study Participation. Fetal Diagn Ther..

